# Macroalgal germplasm banking for conservation, food security, and industry

**DOI:** 10.1371/journal.pbio.3000641

**Published:** 2020-02-14

**Authors:** Rachael Wade, Simona Augyte, Maddelyn Harden, Sergey Nuzhdin, Charles Yarish, Filipe Alberto

**Affiliations:** 1 University of Wisconsin Milwaukee, Milwaukee, Wisconsin, United States of America; 2 University of Connecticut Stamford, Stamford, Connecticut, United States of America; 3 University of Southern California, Los Angeles, California, United States of America

## Abstract

Ex situ seed banking was first conceptualized and implemented in the early 20th century to maintain and protect crop lines. Today, ex situ seed banking is important for the preservation of heirloom strains, biodiversity conservation and ecosystem restoration, and diverse research applications. However, these efforts primarily target microalgae and terrestrial plants. Although some collections include macroalgae (i.e., seaweeds), they are relatively few and have yet to be connected via any international, coordinated initiative. In this piece, we provide a brief introduction to macroalgal germplasm banking and its application to conservation, industry, and mariculture. We argue that concerted effort should be made globally in germline preservation of marine algal species via germplasm banking with an overview of the technical advances for feasibility and ensured success.

## Abstract 2

Seaweed germplasm banking is an important resource for biodiversity conservation, human food security, and industry innovation. This Perspective article maintains that an international, coordinative initiative is needed to fully develop and capitalize on this resource.

## Macroalgae are essential members of marine communities and are no exception to the threats of climate change

Worldwide, biodiversity is declining at alarming rates, resulting in what some scholars are calling the Earth’s sixth great extinction event [1]. The marine environment is no exception, with increasing sea surface temperatures leading to drastic alterations in marine populations, communities, and ecosystems [2,3]. Of particular concern is potential for loss of macroalgae (defined as benthic eukaryotic algae of at least 1 mm in length [4]), which function as ecological engineers [5–9], primary producers [3,10], habitat and structure providers [6], nutrient cyclers, keystone species [11], food and nursery grounds for invertebrates and pelagic organisms, and shoreline buffers from storms [12,13]. Furthermore, macroalgae are a US$11 billion industry as food, animal feed, and fertilizers [14–16]. Seaweeds are under threat from multiple stressors including warming sea surface temperatures, pollution, overharvesting, and other anthropogenic disturbances that have major consequences for the structure and function of near-shore coastal ecosystems [13,17]. Although seaweeds are predicted to function photosynthetically well with increases in CO_2_ [18,19], their distributions within their local communities (i.e., occupied tidal zone) and globally (i.e., latitudinal range) are likely to be impacted by increased temperature, threat of desiccation, and other environmental factors that are predicted to be affected by climate change [18,20,21]. The direst of potential consequences include both localized and complete extinction that in turn results in loss of genetic diversity, ecological function, and services provided by marine photoautotrophs [12,17,20–27]. Furthermore, climate change can open opportunities for increased invasion by nonnative seaweeds [28]. Given the historical importance of the mariculture industry and its continued growth globally, these effects in the coastal environment will become more pressing for humankind.

## Macroalgae aquaculture through history to the present

Wild harvest of macroalgae has a longstanding history in many cultures, particularly in Asia, Polynesia, and South America. Modification and enhancement of wild stocks began as early as the 17th century in Japan. Today, over 200 species of macroalgae are wild harvested for various industries [29], but only a dozen are currently commercially cultivated [15,30,31]. Current global macroalgae production is worth US$11.7 billion annually, with most production taking place in Asian countries [16]. As global aquaculture continues to increase, the blue economy—a term used to describe the use of ocean resources for economic growth—needs to evolve sustainably to support marine ecosystem function and rejuvenation. Understanding trade-offs and synergies between provisions of different ecosystem services and how these vary with scale and environmental context needs to be a priority for future research [32]. Much of the innovation in macroalgae aquaculture optimization comes from Asia, although other international leaders in the industry have begun to explore its use for biofuel production [33–36]. However, there are still several unpredictable challenges to be overcome before the emerging algal biofuel industry is of considerable value and impact worldwide, particularly disease mitigation, ecological impacts of large-scale aquaculture, evolution of regulatory practices, market development, and the optimization and economical production of biofuel from algal polysaccharides [37]. Additionally, the preservation of strains utilized in these industries is an important strategy for maintained productivity and commerce.

## Algal germplasm banking entails long-term storage of different lifestages

Ex situ seed banking preserves the diploid, embryonic stage of the embryophyte plant life cycle. This stage is optimal for preservation because of its evolved traits that facilitate long-term dormancy in natural conditions (e.g., nutrient-rich endosperm, robust seed coat, specific germination requirements) [38]. Conversely, algal germplasm banking (referred to as biobanking elsewhere) more resembles methodology utilized for long-term storage of nonflowering land plants where either spores or haploid gametophytes are stored [39–42]. However, the cells or tissues targeted very much depend on the species, as the longevity of seaweed microscopic stages is related to the seaweed life strategy that will resume development when returned to adequate conditions [43]. For example, the diploid conchocelis stage of *Pyropia/Porphyra* is the optimal stage for its long-term storage [44,45]. Preservation work for other species has focused on spores [42,46,47]. Others are more easily preserved, like *Gracilaria* and *Agarophyton* species that are well maintained as meristems or mature thalli [48]. Live tissue preservation (i.e., liquid cultures in dormancy versus cryopreservation) is of particular use in the context of strain selection and preservation, and therefore mariculture, as it allows for genotyping of fresh material, the crossing of known genotypes, and preservation of desirable strains for additional manipulations, without potentially deleterious thawing measures. Furthermore, the culturing techniques and hatchery methods have been well developed for a variety of species, particularly for economically important species (e.g., *Chondracanthus chamissoi* [49], *Porphyra yezoensis* [50], *Saccharina* spp. [40,51]), but long-term storage protocols are less commonly available. Thus, we recommend that liquid cultures held under dormancy conditions (e.g., reduced light and temperature) be prioritized for long-term germplasm preservation as a methodology that can be more quickly implemented given the current availability of algal preservation protocols.

## Developing stable aquaculture and germplasm banking techniques are essential for food security, conservation, and innovation

As the human population continues to grow, it is important to explore alternative food sources, like macroalgae, for sustainable nutrition. In collaboration with Knorr Foods, the World Wildlife Fund published a 2019 report supporting *P*. *ubilicalis* and *Undaria pinnatifida* as part of a top-50 foods list that people should prioritize eating for a healthier planet and global food sustainability [52]. As reliance on macroalgae for sustenance increases, the necessity to catalog and preserve edible and nutritious species also grows. Germplasm banks represent the opportunity to provide long-term preservation of important strains and diversity of those strains. These banks also have important implications for conservation efforts and, perhaps more importantly, as an optimal economic approach to conservation and restoration efforts [53]. Ex situ embryophyte seed banks as conservation centers are estimated to cost as little as 1% of traditional in situ conservation [54], although it is important to note that this estimate is exclusively for terrestrial systems. With preserved, live samples easily accessible, restoration efforts are made easier. Furthermore, germplasm material can be used as starting points for the generation of optimized lines via both direct and indirect synthetic biology advances [55]. Germplasm collections can also be the lasting references of historical distribution data, particularly in the context of invasive species identification [56]. Lastly, preserving macroalgae strains and cultures provides an incredible source of material for industrial and research applications. In particular, specific species and strains of macroalgae are indicators of pollution and overall water quality [57], and therefore can be used as early warnings of degrading systems. Algae are also raw material for innovations in biofuel refinement, pharmaceuticals, cosmetics, textiles, and animal feed [58]. Strains with complete genotype and phenotype profiles offer great starting points as model systems for numerous research possibilities, including optimization of biorefining pipelines, development of new macroalgal products and byproducts, and exploration of macroalgal applications for climate change mitigation.

Given the breadth in germplasm bank utility and value, one of the greatest challenges is identifying which species to prioritize; with estimates in algal diversity ranging from 72,500 [59] to 170,000 species [60], complete representation is daunting. Because of this challenge, we recommend prioritization in the following three ways: (1) economic importance, (2) ecological importance, and (3) evolutionary importance. Perhaps the most easily prioritized species are those that are currently grown in aquaculture systems. As mentioned previously, many of these species are already cultivated and optimized for aquaculture. However, these efforts may have also resulted in loss of genetic diversity [61–64], which can reduce resiliency to environmental stressors [65]. Elsewhere, species highly utilized are still largely wild harvested; *Macrocystis pyrifera* for alginate isolation and *Ecklonia maxima* for biostimulant production, for example, are still exclusively wild harvested and have yet to have well-developed mariculture methodologies for large-scale farms (R. Marcos, P. del Piedra, pers. Comm.). Although *M*. *pyrifera* has germplasm collections that represent its distribution and genetic diversity in both hemispheres [39,66], a breeding program with prioritized strains is still being developed. Conversely, *E*. *maxima* has yet to be genetically characterized in its limited distribution or developed to any degree for aquaculture purposes. Thus, economically important species should be prioritized in order to not only preserve optimized strains currently used but also conserve their genetic diversity in order to maintain their utility over time. Secondly, species that provide crucial ecological services (and are therefore regarded as foundational keystone species) should be prioritized for germplasm banking. Particularly canopy- and habitat-forming species in the brown algal orders Laminariales and Fucales should be targeted for research and sampling of genetic diversity [67]. Furthermore, those that provide support and structure of benthic communities, such as coralline algae (Rhodophyta) [68], should also receive prioritization for preservation. Thirdly, species that are of evolutionary value should receive attention for germplasm banking; evolutionary importance can be interpreted as those that have broad geographic ranges and/or exhibit warm temperature tolerance and, therefore, resiliency to climate change. Similarly, species that exhibit regular or perhaps even increased growth and fitness in response to increased dissolved carbon availability and decreased nutrient availability [69] should be targeted (e.g., *M*. *pyrifera* [70], *G*. *lemaneiformis* [71]). Although emerging research provides some indication of what species may comprise that category, the literature is still fairly limited, and because of the fine scale and unique combination of stressors that are likely to be experienced, these species are likely to be difficult to identify at this time [18,72]. Lastly, species that are members of current refugia, such as mesophotic ecosystems, should be considered for long-term preservation. Mesophotic systems not only provide connectivity among marine ecosystems, but can also act as refugia, and therefore are centers of biological and genetic diversity [73,74]. Although all three areas of priority have considerable room for growth and advancement via continued research, they are important starting points for germplasm bank development.

## International cooperation and collaboration are necessary for germplasm banking success

Despite a fast-growing interest in commercial aquaculture, corresponding with a rapidly increasing global demand for nutrition supplementation and industry raw material, few researchers outside of Asia have explored sustainable domestication programs, including the creation of macroalgae germplasm banks [31,39,75,76]. Several facilities, such as the National Institute of Fisheries Science–Seaweed Research Center in South Korea and the Ocean University of China and Chinese Academy of Science, have dedicated infrastructure and research to the maintenance of commercially important macroalgal species and cultivars. However, it is likely that these banks, although incredibly useful, are lacking in not only their intraspecific diversity but also overall species diversity. But the burden to prioritize and value macroalgal conservancy should not be placed on these few banks, and there must be international recognition of the importance and utility of macroalgal germplasm preservation. Furthermore, without international buy-in to macroalgae as an important component of mariculture, the global industry faces the steep hurdle of implementing a sound, eco-centric framework with which to grow sustainable and economically viable macroalgae products [77–79]. We suggest the following in order to achieve the initiation of such an international cooperative: (1) Legislative/government officials must support and champion such an initiative at the national level; (2) the cooperative units must agree on at least broad categories, as those suggested previously, of species that should be prioritized worldwide; and (3) the cooperative units must agree to share germplasm collections in order to provide replication and ensure survival of the collections. Although optimized aquaculture strains are usually owned privately, at least replication of wild strains and representation of overall biological diversity should be accepted unanimously. The precedence for resource sharing is set by the Nagoya Protocol, whose obligations described there within, should be upheld and followed for germplasm banks as well. Additionally, suggestions described by the PHYCOMORPH European Guidelines for A Sustainable Aquaculture of Seaweeds (PEGASUS) protocol for regional to national coordination should be considered and implemented to encourage fine scale collections [80]. In particular, initiatives that incorporate buy-in and management by current aquaculturists and professionals working in marine products, such as the small, localized cooperatives that are managed by fishermen seaweed farmers in Japan, should be prioritized as infrastructure as the national level is constructed. In summary, we promote that support and management of germplasm at every level (i.e., local, state, regional, and national) will be essential to the success and longevity of macroalgal germplasm banks.

## The need for genetic profiling for macroalgal germplasm banking and its challenges

A major obstacle to the creation of an extensive and viable macroalgal germplasm bank rests on the documentation and understanding of the genetic variation in coastal macroalgal communities and how genotype influences each organisms’ phenotype and its functional importance within the ecosystem. Maintaining biodiversity within a marine macroalgal ecosystem necessitates observing key interactions within a community to identify those species that provide invaluable ecological services [81] and maintaining that biodiversity and functional variation in macroalgal germplasm banks [82]. Loss of seaweed genetic diversity through poor commercial breeding programs has led to yield declines globally [62,63,83,84]; therefore, maintaining wild or “heirloom” strains is critical for continued yield success of cultured seaweed and for maintaining diversity and ecosystem function globally.

Major challenges of documenting genetic diversity across distributions have included not only considerable collection costs but, historically, impeding sequencing costs as well. However, work done over the past 30 years with increasingly economical sequencing approaches has made important progress in our understanding of genome evolution, genetic diversity, population structure, and ecosystem dynamics in commercially and ecologically important macroalgae (e.g., *Macrocystis* [6,66,85–89], *Porphyra* [62,90–94], and *Saccharina* [40,51,95]). A well genotypically characterized germplasm bank can provide a wealth of information for macroalgal varieties, breeding lines, and has been developed for several strains in Asia and is currently underway at several facilities in North America and Europe [96]. These germplasm collections will serve as a valuable tool both for future conservation and aquaculture efforts and will only increase in impact with increased support for facilities, infrastructure, and breadth of culture collection.

## Conclusions

In closing, we emphasize that international and national programs that are currently supporting terrestrial plant seed banks (e.g., botanical gardens, Global Crop Diversity Trust, Plant Conservation Alliance, Seeds of Success, among others.) should equally support macroalgal germplasm banking efforts. Building a global network and coordinated initiative to build and maintain these collections is vital to supporting the Blue Revolution and continued mariculture advancement. By incorporating macroalgal germplasm banks into these efforts, we support not only human food security and industry but also the ecological function and services provided by marine primary producers. We also recognize that the ideas presented here are not necessarily novel; it is important that recognition of documents like PEGASUS be given. But given the very recent interest in seaweed mariculture outside of Asia and Europe, it is important that conversations surrounding cooperative and thoroughly characterized macroalgal germplasm banking begin as soon as possible, and the ideas presented here continue in their development as a living concept.
